# Multi-Resolution 3D Rendering for High-Performance Web AR

**DOI:** 10.3390/s23156885

**Published:** 2023-08-03

**Authors:** Argyro-Maria Boutsi, Charalabos Ioannidis, Styliani Verykokou

**Affiliations:** Laboratory of Photogrammetry, School of Rural, Surveying and Geoinformatics Engineering, National Technical University of Athens, 15780 Athens, Greece; iboutsi@mail.ntua.gr (A.-M.B.); cioannid@survey.ntua.gr (C.I.)

**Keywords:** augmented reality, multi-resolution, AR.js, Three.js, web visualization

## Abstract

In the context of web augmented reality (AR), 3D rendering that maintains visual quality and frame rate requirements remains a challenge. The lack of a dedicated and efficient 3D format often results in the degraded visual quality of the original data and compromises the user experience. This paper examines the integration of web-streamable view-dependent representations of large-sized and high-resolution 3D models in web AR applications. The developed cross-platform prototype exploits the batched multi-resolution structures of the Nexus.js library as a dedicated lightweight web AR format and tests it against common formats and compression techniques. Built with AR.js and Three.js open-source libraries, it allows the overlay of the multi-resolution models by interactively adjusting the position, rotation and scale parameters. The proposed method includes real-time view-dependent rendering, geometric instancing and 3D pose regression for two types of AR: natural feature tracking (NFT) and location-based positioning for large and textured 3D overlays. The prototype achieves up to a 46% speedup in rendering time compared to optimized glTF models, while a 34 M vertices 3D model is visible in less than 4 s without degraded visual quality in slow 3D networks. The evaluation under various scenes and devices offers insights into how a multi-resolution scheme can be adopted in web AR for high-quality visualization and real-time performance.

## 1. Introduction

Web AR advances along with high-end mobile computing and alters how experiences are interpreted. Common digital overlays vary from text and animations to 3D graphics and low-poly models. However, there is a demand for more intuitive, realistic and efficient types of media in order to increase user engagement and retention. Such examples are the detailed and high-resolution 3D models that are derived from laser scanning, photogrammetry or fast surface modeling. Their geometry is represented as a mesh of triangles, quadrilaterals or other polygons that includes the shape, size and position of the object’s vertices, edges and faces. These mesh data often exhibit spatial redundancies, such as repeated vertex positions or similar attribute values, non-manifold geometry, outliers and surface discontinuities. Besides the inner structure, the accompanied texture maps, material properties, lighting information, animation data, metadata, etc., add to their overall complexity. The process of streaming these assets on the web is subject to a set of physical and logistical constraints: storage, bandwidth, latency, memory and computational power [1]. WebGL graphics API and rendering engines typically expect well-formed meshes with consistent topology. Since there is a lack of 3D web standards, a combination of proprietary formats, third-party libraries and custom workflows tailored to the requirements of the current project is usually adopted. Besides hardware acceleration, common rendering practices include adaptive streaming, progressive loading and optimized compression.

Meanwhile, AR is a motion-tracking issue that requires stable poses and accurate localization. The recovery of 3D poses from a frame sequence captured by a live camera involves many steps of intense processing. Feature detection and matching, feature tracking, robust estimation techniques such as RANSAC and relocalization algorithms can place pressure on the computing capabilities of mobile devices. Besides a high CPU load, the real-time 3D rendering proves to be a resource-intensive, long process, even for 3D models larger than 4 or 5 MB [2]. The absence of a lightweight 3D format or a standardized method on a graphics pipeline can result in a user experience degradation and performance limitations. This may manifest as reduced frame rates, increased latency or the inefficient use of device resources, ultimately impacting the responsiveness and smoothness of AR interactions. Moreover, the meshes used as AR overlays may undergo modifications or simplifications as a trade-off for quick loading times and low bandwidth, resulting in visual degradation, reduced detail or lower fidelity compared to the original ones. Therefore, the AR overlays may not fully capture the intricate details and realism of the original 3D content, affecting the overall experience.

The presented work examines a streaming-friendly multi-resolution scheme for high-resolution textured 3D models in the context of web AR. A prototype that integrates cluster-based view-dependent rendering by the Nexus.js algorithm and allows for the loading of 3D models of relevant NXS and NXZ formats to the AR scene has been developed [3]. Its user interface allows switching between two types of AR: natural feature tracking (NFT) using pre-trained images and relative location-based position accessing the inertial sensors of the mobile device. Parameters related to position, rotation, scale, FOV and camera distance for AR overlays can be adjusted in real time. The implementation entails the AR.js library [4], the built-in artoolkit for tracking [5] and the Three.js 3D graphics library that lies within the WebGL API [6]. The performance evaluation compares and assesses the efficiency of the following, based on specific evaluation metrics such as rendering performance, responsiveness and visual fidelity:

- multi-resolution NXS structures against optimized binary glTF (glb) format using meshoptimizer library [7];
- compressed version of the multi-resolution algorithm (NXZ) against Draco’s structure (DRC) [8].

Each method is incorporated into a Three.js wrapper for runtime rendering on a set of four meshes that covers a range of complexities, sizes and vertex/index data variations. The effects of different parameter values on conversion between formats, compression and optimization processes are recorded and analyzed. In addition, guidelines for the parameter tuning process, to manage redundancies in vertex and index data, which are common in photogrammetric mesh structures, are provided. Finally, a geometry instancing technique that stores the common base geometry once and specifies the per-instance data and transformations separately in AR sessions is developed by extending the Nexus.js codebase. The computational overhead associated with rendering individual instances is minimized, producing more consistent frame rates when a new instance or a new AR type is drawn or enabled, respectively. According to the real-time benchmarking results, CPU consumption is reduced by up to 21% and 46% compared to common glTF rendering and Draco decompression, while the AR experience is excellent, with no loss of visual quality, even for large-volume data exceeding 34 M vertices. Compared to glb, the NXS format results in an average of a 46% speedup in rendering time and up to a 34% smaller GPU load. Draco compression, although resulting in smaller file sizes, introduces longer decoding times, which impact the responsiveness of the AR application, particularly for larger and more complex models. The evaluation of the performance showcases the effectiveness of the view-dependent Nexus rendering approach, demonstrating its suitability as a lightweight AR format capable of handling large, irregular and textured 3D data generated through photogrammetric and computer vision techniques.

The rest of the paper is organized as follows. Section 2 reviews related methods, works and current advancements regarding compression libraries, view-dependent techniques and optimized rendering for web AR. In Section 3, the steps of the proposed methodology are described in detail. Section 4 outlines the implementation and results of the AR prototype. Section 5 assesses the performance of the multi-resolution format (NXS) in comparison with its compressed version (NXZ), DRC and optimized gLTF formats and documents the findings. Finally, Section 6 concludes the proposed work and depicts the future research steps.

## 2. Literature Review

### 2.1. Optimized 3D Rendering

Numerous studies have addressed the optimized web 3D visualization of large and complex geometry. In this section, only view-dependent approaches, able to adapt to the concurrent operation with the already arduous task of AR, and the tile-based rendering of mobile devices are presented and discussed. For instance, progressive mesh streaming prevents geometric attributes but is computationally intensive since every part of the model is rendered if certain criteria or other techniques are not set [9]. Multi-triangulation is constrained by the high CPU load required to traverse the direct acyclic graphs (DAGs) and by the fact that is not possible to fully exploit the GPU processing power [10].

#### 2.1.1. Compression

Compression significantly reduces the file size of 3D models, making them more efficient to store, transfer and render in real-time applications. General-purpose compression libraries, such as Zstd [11] or Oodle [12], target real-time visualization cases where a fast decompression speed is a crucial factor. However, they are not designed to exploit redundancies in vertex and index data and they may result in low compression ratios. Instead of basic mesh decimation and simplification algorithms, which try to reduce the vertex and face count with minimal shape changes [13], dedicated arbitrary geometry algorithms utilize one or a combination of the following techniques: quantization, delta encoding, vertex cache optimization or octree-based encoding. The Draco algorithm primarily adopts quantization and delta encoding to reduce the precision of vertex attributes and encode the differences between the consecutive ones. While it is highly effective at compressing complex meshes with high vertex and triangle counts, it does not prioritize the UV texture mapping quality. Additional compression techniques specific to textures may need to be considered alongside Draco for optimal results, such as Khronos TeXture (KTX2) [14]. Other algorithms convert the connectivity, geometry and vertex attributes into a stream of symbols and bits to reduce the data volume and allow for progressive transmission during decompression [15,16,17]. The decompression time on the client side is of greater importance than the compression rate. In the case of single-rate compression, WebGL-Loader of Google provides a better decompression ratio than the Draco system but they both do not support progressive encoding/decoding [18]. The Corto library compresses large point clouds and meshes with per vertex attributes, aiming to support streaming and adaptive rendering [3]. Regarding decoding, edges are connected in such a way that allows for the efficient traversal and fast reconstruction of the mesh topology, especially when viewing distance or angle-based criteria are employed.

#### 2.1.2. View-Dependent Techniques

A model compression technology can only shorten the download time; the rendering operations on mobile devices still consume a large amount of CPU, memory and battery resources. A natural approach to reducing the model data is a simplification method that represents the models with selective levels of detail depending on their properties or user-defined criteria [19]. Level-of-detail (LOD) techniques can be broadly categorized into discrete and continuous LOD based on the transition between levels, as well as into hierarchical, screen space and temporal LOD if the respective optimization factor is scene hierarchy, screen visibility or temporal coherence. View-dependent LOD considers the viewer’s position and orientation to dynamically select the appropriate LODs, optimizing the rendering for the areas visible to the viewer. Since the display of an overlay is dynamically adjusted based on the viewer’s perspective and the direction of the camera, view-dependent LOD could be a valuable feature for AR. Unlike the view-dependent approaches, the meshoptimizer library reorders triangles to minimize the overdraw from all directions and not only from the point of view [7]. It primarily focuses on mesh optimization, such as vertex cache optimization, index buffer optimization and vertex quantization, rather than a specific loading strategy. These processes help to decrease memory consumption and enhance the rendering performance by minimizing the time required for index fetching. However, quantizing vertex attributes may cause a minimal loss of visual quality.

A hybrid approach that includes the aforementioned techniques should be engaged. Thus, encoding, quantization and compression come as a natural extension to the family of multi-resolution algorithms. Multi-resolution structures for the web have been well studied since 2000 and typically involve representing a 3D model using a hierarchy of levels of detail with varying degrees of geometric complexity [20]. Any type of multi-resolution representation can be naturally extended to a view-dependent streaming geometry due to its intrinsic characteristics [21]. For example, in multi-resolution strip masking, tiles can be rendered at various resolutions, according to their global and local properties, using a set of coarse to fine masks, which are precomputed triangle strip indices [22]. While it effectively offloads a significant portion of the CPU load to the GPU, it is less geometrically optimal than local LOD algorithms. On the other hand, several algorithms have been introduced for cluster-based partition driven by several criteria, such as partition, hierarchy, kernel, quantum theory, etc. [23,24]. Gobbetti and Marton transformed point clouds into two-manifold meshes and encoded them into compact multi-resolution structures consisting of variable-resolution quad patches [25]. The patches store both geometry and texture information in a lightweight, tightly packed texture atlas. These 3D structures have the potential to be employed as AR overlays since the GPU-accelerated adaptive tessellation algorithm of the method minimizes the CPU overhead. On the other hand, the Nexus algorithm adopts a patch-based data structure, from which view-dependent conforming mesh representations can be efficiently extracted by combining precomputed patches [3]. Since each patch is itself a mesh composed of a few thousand triangles, the multi-resolution extraction cost is amortized over many graphics primitives, and CPU/GPU communication can be optimized to fully exploit the complex memory hierarchy of modern graphics platforms. It defines a publishing format (NXS) for multi-resolution meshes that stores the sequence of the increasingly coarse partitions. More recent multi-resolution schemes elaborate their parallel compression algorithm to the GPU for hardware acceleration [26,27]. They achieve significant performance improvements in cases of multi-core computing, currently excluding consumer mobile devices. A hardware tessellation-based patch rendering with continuous LOD is a promising approach for AR, offering a stable frame rate for high-fidelity outputs with optimum memory and computational usage, but it currently addresses triangular models [28].

### 2.2. Web AR Approaches for 3D Overlays

In general, the synergy of open-source JavaScript AR software such as AR.js with the Three.js library has a variety of applications in interior design [29], cultural heritage [30], synthetic visualizations [31] and education [32,33]. Nitika et al. developed a WebRTC marker-based application with AR.js and Three.js to analyze web AR performance, examine potential rendering optimizations and proposing a cloud-based solution [34]. Rendering can be offloaded to cloud servers, minimizing the overhead, but data transfer suffers from E2E latency and the risk of security and privacy threats. MEC-oriented web AR solutions compensate for this latency, as well as the weak computational efficiency of browsers, through the operation of a client–server model typically located close to the user’s device. However, both technologies are prone to single-point failures and network loads while huge or multiple chunks of 3D data are transferred. From a rendering performance standpoint, there is not a lightweight 3D format file suited to overlays, while the emergence of dedicated mobile web AR browsers [35,36] has not resolved the compatibility issues that arise from the lack of a standard for web 3D objects. The Universal Scene Description (USDZ) schema, developed by Apple and Pixar, is a compact format that incorporates AR functionalities into 3D content [37]. Apart from the typical storage of geometry, materials, textures, animations, lighting and camera properties, it defines the state, the tracking mode and the location data of the AR experience. Being primarily designed for the iOS operating system, it is tightly integrated with Apple’s ARKit and has limited authoring tools. This conflict between having a uniform cross-platform SDK or API and leveraging the unique characteristics of each platform is still an open problem [38]. glTF and its binary type glb is a royalty-free specification of the Khronos Group for the efficient loading of 3D scenes on the web [39]. A remaining issue is that the various glTF renderers do not always appear to produce visually identical results [40]. Apart from the format, large-sized and detailed source data of arbitrary geometry require pre-processing or further optimization steps at runtime by the rendering engine or the framework. gltfpack of the meshoptimizer suite [41] applies a set of optimization steps, including encoding, reordering, quantization and memory management, to the main GPU procedures (vertex processing, rasterization and fragment shading), while the Gltf pipeline by CesiumJS [42] supports quantization and Draco compression.

Only a few approaches can be considered complete regarding remote 3D rendering during an AR session. A dynamic adaptive streaming technique represents AR assets at different LODs and only the LODs of high fidelity are selected by a heuristic to be visualized each time. It manages to decrease the start-up latency by up to 90% with respect to a download-and-play baseline, as well as the amount of data needed to deliver the AR experience by up to 79%, without sacrificing the visual quality [43]. Another work dedicated to the rendering phase of augmentation, developed for Mobile AR (MAR), exploits a meta-file during the progressive HTTP streaming to determine the next requested chunk of data and enhance the visual quality of the view-dependent display [44]. An HTTP adaptive AR streaming system that aims to provide high-quality AR streaming services with low latency over time-varying wireless networks has recently been introduced [45]. The system utilizes progressive mesh techniques coupled with a metafile structure to render, display and schedule the streaming of chunks of data according to the wireless network load. Unlike the view-dependent rendering of Nexus.js, it adapts to different network conditions, and it takes into account the displayed scale values of the AR overlays on the screen, ensuring that the requested chunks of content are optimized to enhance human visual perception.

This work offers insights into how a multi-resolution scheme can be embedded in web AR to provide high-quality visualization and real-time performance for large-sized and complex 3D models. It extends the capabilities of existing frameworks such as AR.js and Three.js, integrating them seamlessly to handle a multi-resolution 3D format and enable user-friendly interactions and parameter adjustments in real time. The choice of AR.js is made because the underlying AR library accounts for its compatibility across browsers and devices with WebGL and WebRTC features [46], while the selection of the Nexus.js algorithm lies in its proven capability to handle arbitrary geometry [47,48], its good compression ratio and its fast decoding (3 M triangles per second with normal and colors in a single thread) even at low-end devices. By comparing some of the aforementioned notable rendering schemes and their respective advantages and limitations with the proposed one, it is shown that the suggested method achieves progressive streaming and the responsive view-dependent visualization of textured high-resolution 3D models of a large size (more than 1 GB) and data volume. It also outperforms the traditional Draco compression–decompression and glTF loading approaches in terms of efficiency and performance. The presented research contributes to the progress of web AR by providing a comprehensive and efficient solution for the rendering of high-resolution 3D overlays in augmented reality experiences.

## 3. Methodology

The method consists of three phases: the offline conversion of the 3D models into multi-resolution structures of NXS and compressed NXZ format, the runtime rendering into a Three.js wrapper and the regression of their 6-DOF pose. The progressive loading and view-dependent resolution achieved during the AR session is based on the Nexus algorithm, which is described in [16]. An overview of the algorithm is provided for the completeness of the paper. Then, the developed AR scenarios, including NFT and IMU-based relative location, the rendering process and the geometric instancing technique for the final visualization of multiple meshes on AR, are analyzed. Finally, the Draco compression logic and structure, applied to the data for testing and performance comparison purposes, is briefly presented.

### 3.1. Multi-Resolution Nexus.js Scheme

The Nexus.js algorithm subdivides the mesh into a sequence of increasingly coarser partitions, interleaved with simplification steps. This process is defined as Batched Multi-Triangulation (BMT), which relies on the concept of V-partitions [49]. Let *H* be a partition of a model *M* into n disjoint regions *h*_1_, *…*, *h_n_* and *G* = *g*_1_, *…*, and let *g_t_* be a partition of the same model into t disjoint regions. We denote with H∩G the intersection of the two partitions defined as

(1) $$H\cap G=\underset{i=0\ldots n,j=0\ldots t}{⋃}\left\{h_{i}\cap g_{i}\right\}$$

**Figure 1:**
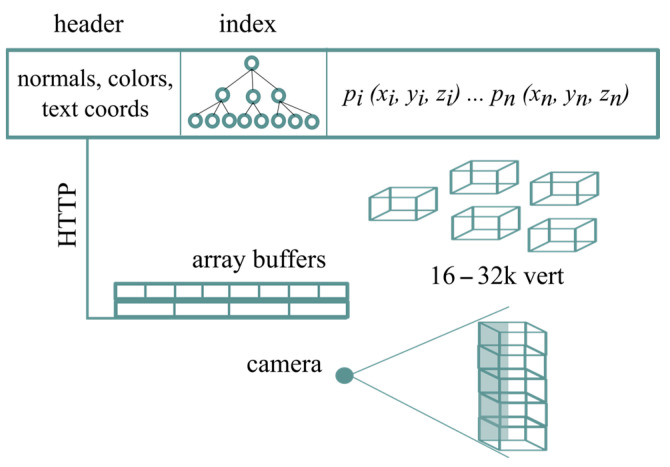
Illustration of the structure and streaming process of the multi-resolution structure of the Nexus.js algorithm.

The construction of a multi-resolution structure over a model M starts with the definition of a sequence of coarser partitions *H*_0_, *…*, *H_n_*. In the subsequent step, the partition $L_{0}=H_{0}\cap H_{1}$ is created by intersecting the two finest partitions and splitting the model into patches, one for each cell of *L*_0_. Multi-resolution 3D models are typically stored in file formats such as VRML, X3D or COLLADA, which support the storage of multiple levels of detail for a single model. The data structure of the presented algorithm is NXS. It is composed of a header with the attributes of the model, an index with the tree structure of the patches, the vector with the positions of the patches and the patches themselves (Figure 1). Each volume partition is defined by a KD-tree built on the triangles of the model. HTTP range requests download the header and the index and array buffers parse the structure into JavaScript. The patches are then downloaded, prioritizing the highest screen errors.

The rendering requires the traversal of the patch tree, computing the approximated screen space error in pixel from the bounding sphere and the quadric error (or any other error metric) during simplification. The traversal is stopped whenever the triangle budget is reached, the error target is met or the required patches are still not available. The main challenge is to preserve the boundary vertices while blocks from different levels are joined in the hierarchy. Once the first patch is downloaded, rendering can start and the model is refined as soon as the rest of the patches are available. Nexus.js supports the compression of the multi-resolution NXS files into NXZ files, through the Corto library. The decoding process in the Corto library at runtime utilizes edge-based decoding, which represents the mesh using a set of directed edges (DEdge2 objects) stored in the front vector. Each edge corresponds to a triangle face in the mesh, and the edges are connected in such a way that allows for the efficient traversal and reconstruction of the inner topology. It also exploits a form of predictive encoding, where the vertices of the mesh are predicted based on previously decoded vertices and encoded differentials. This prediction is achieved through an array, which stores face information used for vertex prediction.

### 3.2. Pose Estimation

#### 3.2.1. Natural Feature Tracking

NFT is a pattern-based tracking method that involves identifying distinctive features in the live camera feed that are invariant to changes in rotation, scale and lighting. They are extracted once in the pattern image (offline) and in every camera frame during runtime. The offline process includes detecting and extracting feature points from the image that will be used as NFT markers into three files. AR.js uses the Features from Accelerated Segment Test (FAST) corner detection algorithm to identify key points, i.e., areas of high contrast or distinctive patterns, and the Binary Robust Independent Elementary Features (BRIEF) descriptor for the compact representation of the detected key points. The generated files encode the information needed to match the detected features during runtime. They contain the feature descriptors for each key point in the image and their 3D coordinates, used for pose estimation and index mapping between the feature sets and the original image.

In the already defined scene, the 3D model is loaded and stored into a variable. The current renderer has the same size as the browser window and the background alpha value is set to transparent on top of the camera view. An event listener is delegated to the display window to check for resizing and rotating. Tracking detects at 30 frames per second. Once jsartoolkit5 is initialized, features and descriptors from the current frame are extracted. The detection algorithm in jsartoolkit5 is based on a combination of the Kanade–Lucas–Tomasi (KLT) feature tracker and a square marker shape detection algorithm. It then compares these descriptors to those stored of the files. If a sufficient number of matches is found, the position, orientation and scale of the 3D model, namely its pose, are estimated and an event is triggered. The rotation vector of the 6-DOF camera pose is converted into a 3 × 3 rotation matrix using the Rodrigues formula and the result of this computation is the joint rotation–translation matrix [*R|t*] for each frame. It expresses the translation of the origin of the world coordinate system into the projection center, which is the origin of the 3D Cartesian camera system, and the rotation of the world coordinate system into the camera system. The mathematical model used is the projection transformation, which is expressed by Equation (2):

(2) $$\lambda \left[\begin{array}{c} x\\ y\\ 1 \end{array}\right]=\left[\begin{array}{c} c\;\\ 0\;\\ 0\; \end{array}\begin{array}{c} 0\;\\ c\;\\ 0\; \end{array}\begin{array}{c} x_{0}\\ y_{0}\\ 1 \end{array}\right]\left[\begin{array}{c} r_{11}\;\\ r_{21}\;\\ r_{31}\; \end{array}\begin{array}{c} r_{12}\;\\ r_{22}\;\\ r_{32}\; \end{array}\begin{array}{c} r_{13}\;\\ r_{23}\;\\ r_{33}\; \end{array}\begin{array}{c} t_{1}\\ t_{2}\\ t_{3} \end{array}\right]\left[\begin{array}{c} X\\ Y\\ Z\\ 1 \end{array}\right],$$

where *x*, *y* are the image coordinates of a key point in the camera frame corrected by the effects of distortion; *X*, *Y*, *Z* are its real-world coordinates; *c* is the camera constant; *x*_0_, *y*_0_ are the coordinates of the principal point; *r_ij_* are the elements of the rotation matrix *R*; *t_i_* are the elements of the translation vector *t*; and *λ* is a scale factor.

The jsartoolkit5 tracking state is pushed into the render queue and the mesh is displayed in the center of the pattern object, with the default pose. Pose parameters are also sent to the render queue and the animate function is called to render every frame. The browser calls “requestAnimationFrame” at 60 fps to repaint before the screen is drawn. The following pseudocode describes the algorithm for NFT-based pose estimation using jsartoolkit5′s tracking, resulting in the normalized direction vector (Algorithm 1).
**Algorithm 1** NFT-based pose estimationinput: *s* = scene    *c* = camera    *r* = renderer   *imdes =* descriptor/pattern 1: *Ar* = ARsession (*s*,*c*,*r*) 2:  **for**
*trackingbackend*
**do** 3:      onRender.push(*Ar*, *imdes*,) 4:        **if** imdes > tolerance 5:         keyframe = *calculateKeyframe* [*R|t*], 6:         anchor = *createAnchor*(*x, y*); 7:        **else** 8:         continue *trackingbackend* 9:   **end for** 10:    GLTransformation (*x*, *y*, *keyframe*)  output: normalized direction vector

#### 3.2.2. Relative Location-Based

To achieve marker-less AR and indoor positioning, the AR.js library can track the camera’s position and orientation through the device’s inertial measurement unit (IMU) sensors without the need for a physical marker. The 3D model is overlaid in a relative position and by a certain pose a short distance away from the camera. This scenario allows for a flexible and versatile user experience in situations where the end-user’s location is not directly relevant to the information or experience being provided, or in cases where the physical context may be undesirable or inaccessible (e.g., dangerous or inaccessible in the real world). Moreover, by eliminating the dependency on feature-based tracking, relative positioning suits cases where the physical environment lacks distinct features or where the user wants to interact with AR content in locations that are not predefined or previously mapped.

The developed marker-less AR and indoor positioning through relative positioning and IMU tracking aligns with current advancements in the field and is a long-established technique for localization. Inertial sensor measurements have high sampling rates, allowing for precise tracking of the device’s movement and changes in orientation [50]. They are coupled with camera motion’s estimates to partly resolve the scale ambiguity, to provide motion cues without visual features, to process more features and to make the tracking more robust in a wide variety of AR applications [51]. However, they are prone to integration drift, which means that errors in the sensor measurements accumulate over time, leading to inaccuracies in long-term position and orientation estimation. Thus, sensor fusion is applied to enhance the accuracy and reliability of the position and orientation estimates. The libraries used for the prototype provide native functions that fuse accelerometer and gyroscope sensor data with the magnetometer and the GPS to estimate the device orientation. The “DeviceOrientationControls” object of Three.js tracks the device orientation, obtains the orientation values from an API that accesses the hardware, i.e., alpha, beta, gamma from the accelerometer and magnetic field sensors, and updates the object’s rotation based on the device’s movement. Then, the initial position of the camera is set, based on the device’s geolocation using the “setWorldPosition” method, which takes longitude and latitude values and converts them to the world coordinate system. Then, the mesh is positioned relative to the end-user by adding a small offset (long + 0.003, lat) to position the mesh slightly ahead in the scene, aligned with the camera’s forward direction. The “update” method is continuously called within the render loop to ensure that the camera’s rotation is updated based on the device’s movement. Thus, the camera’s rotation is updated based on the device’s movement, reflecting any changes in the AR scene. However, the dynamic measurements of the inertial sensors, namely time lag and drift over time, while the device is moving or being reoriented at some reference position, are not considered.

### 3.3. Visualization and Geometric Instancing

The multi-resolution algorithm draws a geometry in the WebGL API at the appropriate resolution given the point view. After defining the WebGL context, the NXS/NXZ model and its attributes (normals, colors, UV texture and faces), the rendering function is called. A program is needed to bind the nexus.js renderer and then create a scene to set up loading and visualization. The Three.js library is a higher-level abstraction over WebGL and provides features such as camera controls, lighting, materials and 3D object loaders. Custom shaders are created by writing the vertex shader and the fragment shader and assigning the two programs to the material. Regarding the presented work, the following rendering techniques and loaders have been developed and integrated into the AR prototype.

**Figure 2:**
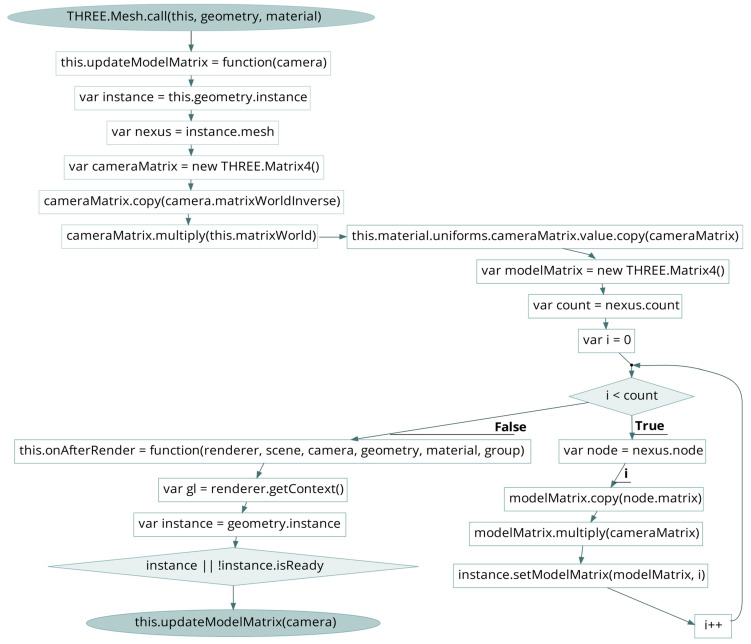
Code snippet with the “updateModelMatrix” function that calculates the joint rotation–translation matrix [*R*|*t*] of the camera pose by combining the camera’s world inverse matrix with the NexusObject’s world matrix.

The dedicated functions that define the multi-resolution rendering context, extend the mesh class of Three.js and load the NXS model are briefly described below. The constructor of the multi-resolution structure initializes the instance of the geometry buffer and a float array with the position of the geometry. The class “Nexus.Instance” creates the WebGL program and reads the specified path model path. When the mesh is loaded, the center and radius of the mesh’s bounding sphere and bounding box are calculated. Then, the potential attributes of vertex, index, UV texture coordinates and material are assigned to the scene instance. Since, in AR sessions, four different 3D models are overlaid, switching from one tracking mode to another, each instance of the same model is rendered with different world transformations and view matrices depending on the current pose. Instead of duplicating the geometry data for each instance, the common base geometry can be stored once, and the per-instance data can be specified separately. Geometry instancing is a technique that involves rendering multiple instances of a base geometry efficiently with different properties or transformations, reducing the number of draw calls and the memory footprint. It is applied to the prototype, to the loader of Nexus.js, extending its native codebase. In Three.js, this is achieved using the “InstancedBufferGeometry” and “InstancedBufferAttribute” classes. After copying the vertex, index, normal and color data from the Nexus instance geometry to instancedGeometry’s attributes, the current joint rotation–translation matrix [*R|t*] of the camera pose is computed by the “updateModelMatrix” function. The developed function combines the camera’s world inverse matrix with the NexusObject’s world matrix. The resulting matrix is passed to the custom shader program as a uniform variable. By default, the “onAfterRender” native function of Nexus.js updates the view of each instance, obtains the WebGL program and sets the attributes and the uniforms of the mesh. By incorporating the camera pose into the model matrix of each instance, the function is modified to ensure that the instances are positioned correctly in the scene relative to the camera’s pose. This allows for the accurate rendering and alignment of the instances with the camera view in AR mode. Thus, inside the “onAfterRender”, the “updateModelMatrix” function is called to update the model matrix for each instance of the geometry. The updated model matrix is then set for each instance using the “setModelMatrix” function provided by the “Nexus.Instance” (Figure 2). In the context of the 3D scene, the position and scale of the NXS/NXZ mesh are set up based on the dimensions of its bounding box, which can be also adjusted dynamically on visualization via AJAX requests and PHP server-side operations. This pipeline effectively results in a progressive visualization with high granularity for the 3D overlays of both NFT-based and location-based augmentation scenarios. The cache of Nexus.js controls the amount of geometry rendered in order to maintain a frame rate above the target of 40 fps.

### 3.4. Draco Compression

**Figure 3:**
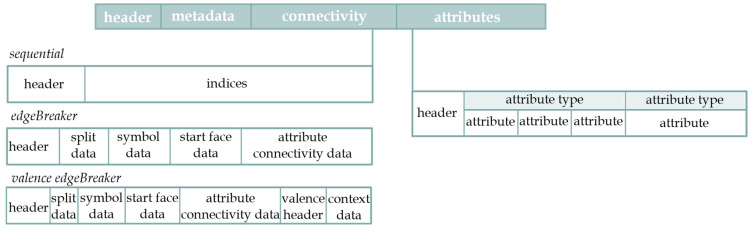
Illustration of the structure of the Draco-encoded geometry (DRC) format.

Draco is an open-source library that uses a combination of compression algorithms, primarily designed to reduce the storage size or transmission bandwidth. Therefore, it maximizes the compression ratio at the cost of disturbing the vertex/index order. It uses quantization and delta encoding techniques to minimize the precision of vertex positions while maintaining visual fidelity. It also encodes the topology of the mesh, which defines how the vertices are connected to form triangles or polygons, resulting in further compression. In addition, DRACO supports the encoding of various mesh attributes, such as colors, texture coordinates, normals and custom attributes. Once a mesh is compressed, it can be stored in a compressed format, such as the Draco-encoded geometry (DRC) format (Figure 3). However, it is important to strike a balance between the compression ratio, decompression speed and visual fidelity when using Draco or similar compression libraries. Quantization bits for primitives such as the position attribute, normal attribute, texture coordinate, color attribute and skinning attribute (joint indices and joint weights) and adjustment of the compression ratio may be applied during conversion.

## 4. Implementation

The developed prototype supports two types of AR, location-based and NFT, and is tested with four meshes of various geometric characteristics, levels of detail and sizes. This section presents the system architecture, the user interface and the results of the AR sessions, as well as the test datasets, their conversion process to multi-resolution structures and their compression.

### 4.1. System Architecture of AR Prototype

**Figure 4:**
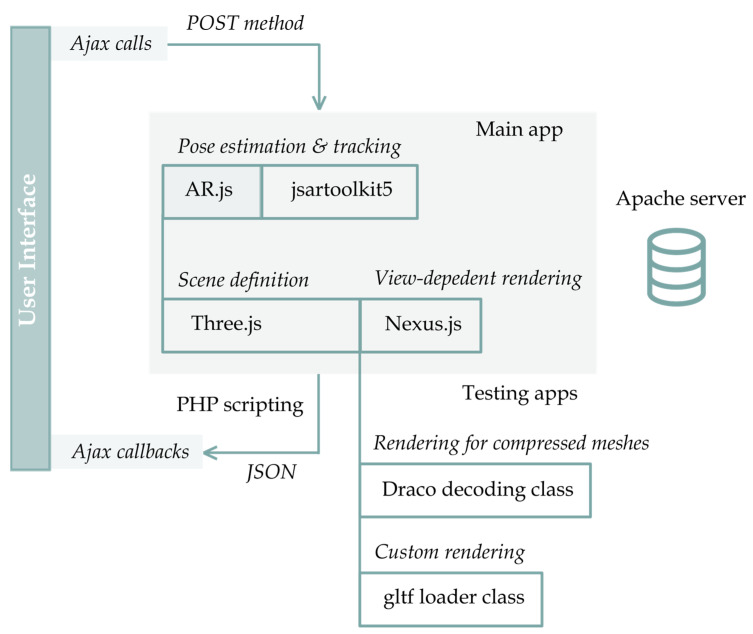
System architecture diagram of the main and testing prototypes.

The web AR prototype is built upon the open-source AR.js library, which features both feature tracking and location-based experiences, as well as jsartoolkit5 for tracking. AR.js and Nexus.js extend objects and functions of the Three.js graphics library for runtime multi-resolution rendering, pose estimation, loading on camera feeds, registration and tracking. The programming toolkit comprises standard web development languages such as HTML, CSS and JavaScript, along with pure PHP scripting and AJAX to handle server-side logic (Figure 4). The prototype integrates a responsive graphical user interface (GUI) that allows it to (i) switch between the two AR tracking types, (ii) select the NXS/NXZ mesh to be superimposed on the real scene and (iii) retrieve, modify and preview the camera parameters as well as the position and rotation of the current mesh, in real time. To achieve this functionality, relevant functions are mapped from the Nexus.js native code using PHP scripting and AJAX on the Apache server. Various PHP scripts compute the bounding box, access the device orientation controls or dispose of the current mesh, handle the AJAX requests and perform the necessary server-side operations. For performance testing purposes, an instance of the DRACO loader class is created in a Three.js scene with the decoder path and then the Draco-encoded geometry is assigned to a 3D object and a buffer geometry. Finally, the glTF loader of Three.js handles the asynchronous loading process of glb files and ensures that they are fetched and parsed correctly. Textures are loaded also asynchronously and applied to the corresponding materials. Regardless of the technique, the loaded Three.js meshes are added to the AR scene with the property of their local path, using AR.js.

### 4.2. Test Datasets and Conversion Process

The input data are the following four models: (i) Middlebury TempleRing [52]; (ii) the Human of CoRBS dataset [53]; (iii) the Anton Memorial of Harvest4D consortium [54]; and (iv) the Meteora site by the Meteora Project [48]. Table 1 shows the format, size and geometric attributes of the 3D models used as AR overlays, before and after their conversion into multi-resolution structures. The Middlebury TempleRing, Anton Memorial and Meteora meshes are derived from image-based photogrammetry and computer vision processing, including SfM, dense point cloud creation, multiple view stereo (MVS) and floating-scale surface reconstruction (FSSR).

Since image-based reconstruction techniques are sensitive to noise, outliers and inaccuracies in the case of complex geometries, the resulting meshes have parts of irregular topology, which manifest as non-manifold geometry and inconsistent triangle connectivity. The 3D files are converted into the multi-resolution format of the Nexus.js library without any pre-processing, to determine how the multi-resolution conversion manages an inconsistent topology. To ensure that intricate details, sharp edges or fine textures are accurately represented in the resulting multi-resolution meshes, Table 2 shows the parameters that are adjusted during conversion. Their choice is driven by the goal of optimizing the computing and rendering performance for AR, after thorough testing, combination and experimentation. The values of the final parameters that attain a balance between file size reduction and visual quality preservation for each model and yield satisfactory results are selected and presented below.

**Figure 5:**
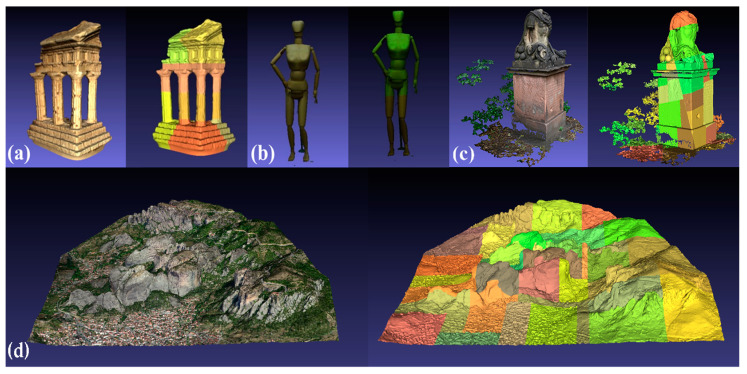
Final NXS meshes and patches visualization of 3D models of varying size and complexity: (**a**) Temple; (**b**) Human; (**c**) Anton; and (**d**) Meteora.

Node faces determine the number of faces per patch in the multi-resolution structure and control the granularity. Small values allow for a smoother change in the resolution of the model. However, larger values are set to balance between performance and visual quality. With larger values, the number of patches in the multi-resolution structure decreases, enhancing the overall responsiveness of the AR session as the server-side processing and data transfer overhead are reduced. Decimation indicates the method of simplification, which is set to quadric. Each vertex of the mesh is associated with a quadric error metric, computed based on the geometric properties of the surrounding vertices and faces. Thus, the simplification algorithm can prioritize preserving important geometric features and maintaining the overall shape of the mesh. The scaling factor controls the level of detail and resolution at each level (default value is set). The adaptive splitting of nodes optimizes the distribution of detail across different levels of the mesh and is set to 0.233 in the case of Meteora, to provide better fidelity for intricate parts of it. Figure 5 shows the final NXS structures with their patches.

Then, the NXS models are compressed using the Corto.js library. The values of the vertex quantization, the quantization factor and the texture compression are adjusted iteratively until the optimal combination that achieves the desired trade-off between compression and fidelity is achieved. Vertex quantization reduces the precision of vertex attributes (e.g., position, normal) to fewer bits, thereby reducing the memory usage and size. The models of Anton and Meteora contain intricate details; thus, the vertex quantization value is set to 6. The quantization factor determines the trade-off between compression and accuracy. A higher factor leads to more aggressive compression but may lead to the excessive loss of details. Since the Human model has fewer details than the others, the factor is set to 0.4. The default value of texture bits is 0.25 but is set to 0.80 to preserve the original resolution (Table 3).

### 4.3. Results

**Figure 6:**
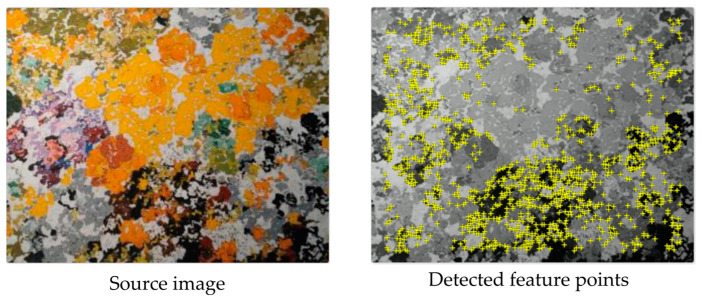
Source image for NFT and detected feature points based on corner detection.

The AR functionality entails the registration, i.e., the spatial alignment, of the real and virtual objects. For marker-less AR based on NFT, the 3D model is displayed in the center of the pattern object, with the Z axis in the direction of the camera and either with a default scale (its greatest dimension being equal to the half of the greatest dimension of the pattern image) or with a user-specified scale. The source image for NFT-based AR is a mousepad with dimensions of 280 × 234 pixels, in which a sufficient number of feature points (439) are detected (Figure 6).

**Figure 7:**
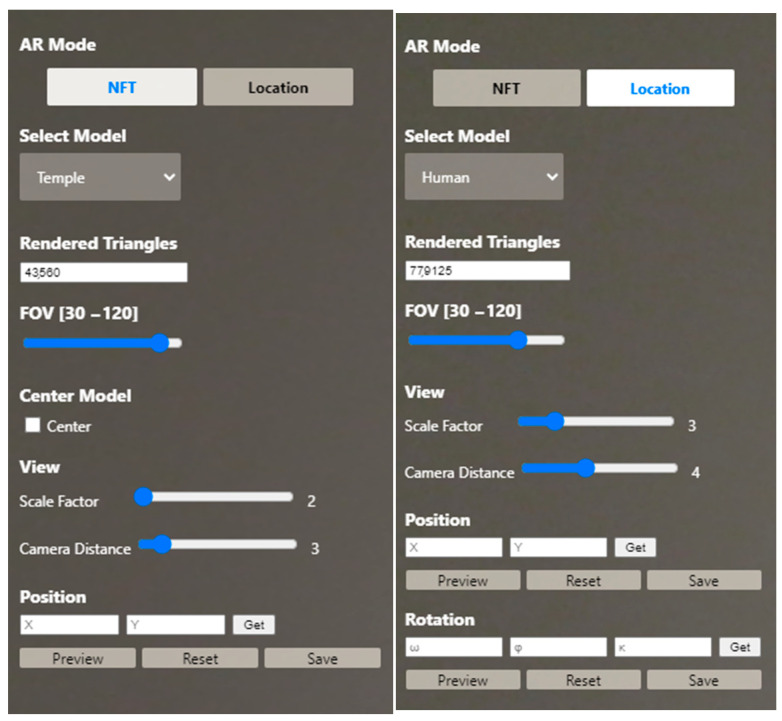
Options related to the manipulation and control of the 3D models in the AR scene in the side panel of the prototype: Case of NFT-based AR (**left**) and case of location-based AR (**right**).

**Figure 8:**
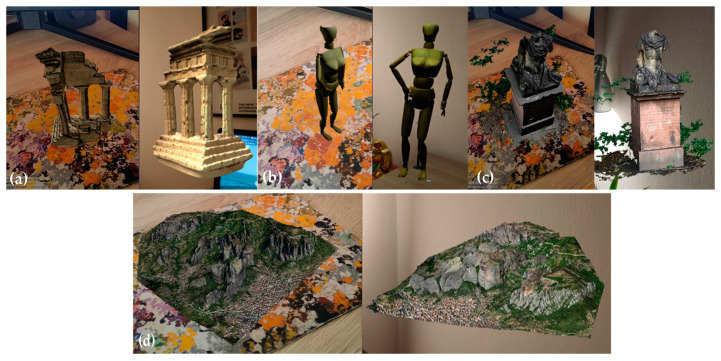
NFT-based (**left**) and location-based (**right**) AR sessions of the prototype with the NXS models: (**a**) Temple; (**b**) Human; (**c**) Anton; and (**d**) Meteora.

In the case of relative positioning, the visualization occurs in relation to the current position of the end-user. By setting a default location of 0.003 degrees of latitude north of the camera, the end-user will face the 3D overlay (Figure 7). The AR prototype contains a side panel on top of the camera feed that enables the control and modification in real time of the following parameters (Figure 8).

1. **AR mode:** Type of registration, switching dynamically between the AR sessions.
2. **Select model**: Drop-down list with the four meshes of the dataset in NXS or NXZ format that can be overlaid on the AR scene.
3. **Rendered triangles:** Number of tringles rendered on the current view
4. **FOV and center mode**: The depth limits are defined with the field of view (FOV) and the aspect ratio, while “center mode” translates the overlay to the center of its bounding box. The “center mode” option is available at NFT-based AR (Figure 8 left).
5. **View:** Adjusting the scale factor of the model and the distance between the camera and the model in the AR scene.
6. **Position**: Retrieving the current *X* and *Y* position values in the AR scene, previewing the model, resetting its position or saving the changes.
7. **Rotation**: Retrieving the current *ω*, *φ* and *κ* rotation values by the inner sensors of the device through the device orientation controls, previewing the model, resetting its rotation or saving the changes (Figure 8 right).

## 5. Evaluation

The prototype integrates the rendering module of the Nexus.js algorithm into NFT and location-based AR sessions and its performance is compared against Draco decompression and common rendering with optimized binary glTF (glb) files. The compression and conversion of the original models into DRC and glb formats, respectively, along with the tuning options and adjustments, are presented, before proceeding to the experimental setup and the benchmarking results. Finally, the results are discussed and conclusions are drawn regarding the potential of the Nexus.js multi-resolution format for web AR.

### 5.1. Comparative Data

#### 5.1.1. Draco

The original models of OBJ and PLY format are converted offline into DRC structures with the encoding tool of the library. The encoder allows various parameters to be modified, such as the compression ratio, quantization bits and texture precision bits. Higher compression levels (up to 10) generally result in smaller file sizes, while lower levels lead to a compressed mesh with potentially better visual quality. The initial size of the Meteora model is 962 MB and, thus, a compression ratio of 8 is applied. A compression level of 0 applies sequential encoding and preserves the face order and is applied to the Temple model. Quantization controls the number of bits used to set the precision of position attributes. Increasing the quantization bits can improve the visual quality but may increase the size of the compressed file. The Meteora model is encoded with the highest value to compensate for the high compression level. The maximum number of quantization bits for texture coordinates is applied to the Meteora model for the more precise representation of texture coordinates. The final size reduction is over 30 times (Table 4).

#### 5.1.2. Optimized glTF

**Figure 9:**
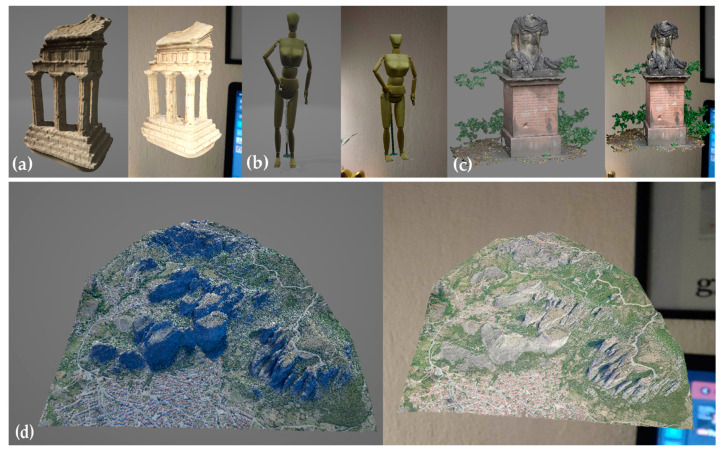
The glb models, (**a**) Temple, (**b**) Human, (**c**) Anton and (**d**) Meteora, on a 3D viewer (**left**) and during the location-based (**right**) AR session of the prototype.

glTF is a compact and streamlined format that represents data with JSON or binary files (glb), resulting in smaller mesh sizes compared to other formats. It is extensively used for the transmission and loading of 3D models on the web, as it has been designed to mirror the GPU API data as closely as possible. As with OBJ and PLY, texture files are loaded at runtime as external image formats. The original models are converted into glb files exploiting the gltfpack library [40] of the meshoptimizer suite. The conversion entails default optimization processes such as the quantization of vertex attributes (e.g., position, texture coordinate, normal, color) and vertex cache optimization, which reorders vertices to improve locality and minimize cache misses during the GPU’s vertex processing. The parameters mentioned in Table 5 are adjusted in order to handle local redundancies and preserve more details. The number of bits used for the quantization of every attribute should be between 1 and 16. The default value for positions is 14 bits and is increased to 16 in the case of Anton and Meteora for higher precision in representing vertex positions. Normals and tangents, which are important for shading and lighting calculations, are quantized with a default of 8 bits. The value is decreased for smaller file sizes. Finally, to preserve more color information, the default value of 8 bits is increased to 14 bits for the Temple and Anton models. A further step of simplification is applied to the Meteora model in order to reduce redundant vertex and index data. It simplifies it based on a target triangle count of between 0 and 1. The option to lock border vertices is activated to avoid gaps in connected meshes and preserve topological consistency. Figure 9 shows the appearance of the glb models in the context of a 3D viewer and as AR overlays on the prototype.

### 5.2. Experimental Setup

The rendering performance, the network load and the memory consumption are evaluated over two AR cases, (i) NFT-based and (ii) marker-less, for the four models separately. The multi-resolution NXS structures are compared against the optimized binary glTF (glb) format and the multi-resolution compressed NXZ structures against compressed DRC structures. All the tests are executed on a high-end smartphone with a Snapdragon 720 G CPU at 2.30 GHz, with 8 GB of RAM, a resolution of 1080 × 2400 pixels and a 60 Hz refresh rate. Network throttling is used to analyze the performance in slower network profiles while each testing session is loaded after cache emptying and a hard reload. The JS execution time is calculated using the “PerformanceObserver” interface of Mozilla Developer Tools, which provides markers to track multiple entries and measure them independently. Performance metrics regarding CPU usage, GPU load, memory allocation, I/O operations, etc., are collected with tracing on the testing device and analyzed on Perfetto.

### 5.3. Benchmarking Results

#### 5.3.1. Runtime Performance

The runtime performance is recorded five times for each 3D model, testing each rendering technique on both NFT-based and location-based AR. Table 6 and Table 7 present the average values of the execution times for the rendering and final display of the overlay, starting from pattern detection in the case of NFT and from the first call of the gpsupdate() function in the case of location-based AR. Regarding the compressed formats, the time elapsed since decoding completion is measured. The noticed difference in performance measures for different inputs is attributed to the inherent characteristics in the input data, the network conditions and the specific rendering techniques used. To account for the variations in performance and understand the degree of variability observed, the standard deviation of metrics is calculated, as expressed in Equation (3).

(3) $$DEV(x_{n})=\sqrt{\frac{1}{n}\sum_{i=1}^{n}{\left(x_{i}-\mu_{i}\right)}^{2}}\;$$

where *N* = number of tests; *x_i_* = detected points in the keyframe; *μ_i_* = mean for each test.

As expected, larger models generally require more time for the final display on the AR scene. In the case of NFT, rendering occurs during the parallel intense tasks of feature detection and image matching, which should be accomplished in the shortest time possible. Thus, the delays between rendering and visualization for location-based AR are shorter compared to NFT. The multi-resolution rendering scheme consistently outperforms glTF loading and Draco decompression under any situation. The display of the AR overlays is significantly faster. At 4.8 s (for NXS) and 4.23 s (for NXZ) from detection and pose estimation, a rough version of the Meteora model (chunk of 154 MB) is already visible in the camera frame. A visual representation to the end-user is provided without any apparent latency, while the finer details and higher resolutions are loaded in the background through progressive streaming. For location-based AR, the time elapsed until the visualization is even shorter, requiring only 4.5 s for the NXS and less than 4 s for the NXZ. Compared to glb, the NXS format results in approximately a 45.18% speedup in rendering time for the Temple model, a 49.26% speedup for the Human model, a 41.28% speedup for the Anton model and 51.79% for the Meteora model. Draco compression generally results in smaller file sizes but longer decoding times compared to the non-compressed formats (nxs. glb). The decoding process of Draco is significantly slower compared to the Corto decompression/decoding algorithm, affecting the responsiveness of the prototype. In the case of the Meteora model, the prototype crashes, caused by an error that occurs during the decoding process. It appears to be to be related to memory management as it indicates an array index out of bounds.

#### 5.3.2. Memory Allocation of Compressed Models

**Figure 10:**
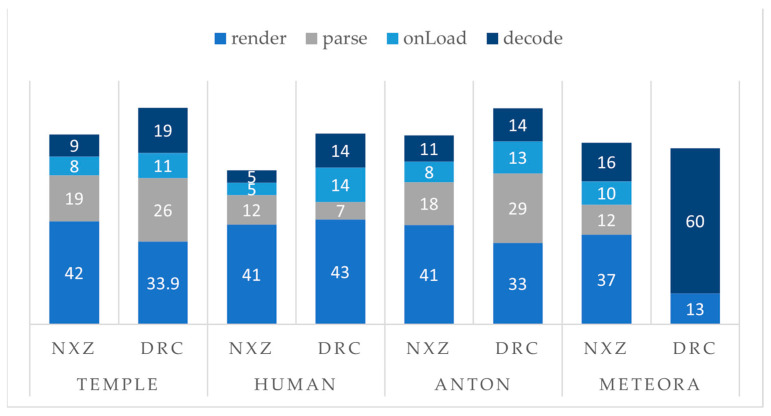
Memory allocation (%) for rendering, parsing, loading and decoding of the models of NXZ and DRC format.

Figure 10 presents the memory allocation results of sampling as percentages of the main functions of the graphics thread, namely render(). getURLparameters() for NXZ or parse() for DRC models and decode().

The render() function consumes a significant amount of memory across all models and compression formats, ranging from 33% to 43%. While the Nexus.js algorithm may optimize memory usage by adjusting the level of detail based on the viewer’s perspective, the actual rendering process itself can still consume a significant amount of memory. This is primarily due to the complex nature of 3D models. Rendering intricate and detailed 3D scenes demands sufficient memory resources to transmit, decode, select with view-dependent criteria and process the necessary information. In the case of Meteora, the error during Draco’s decode function lies in memory leaks and undesirable memory allocation patterns. Even if the compressed DRC model (63.8 MB) is approximately 93.3% smaller in size compared to the initial model, it cannot be handled by the algorithm. The size may exceed the available memory or cause excessive processing time; thus, additional performance factors are seen. Since NFT-based AR involves marker-less tracking and continuous image frame processing and runs simultaneously with graphics tasks, which impacts the CPU load, these AR sessions are further benchmarked.

#### 5.3.3. Frame Rates and CPU Usage

**Figure 11:**
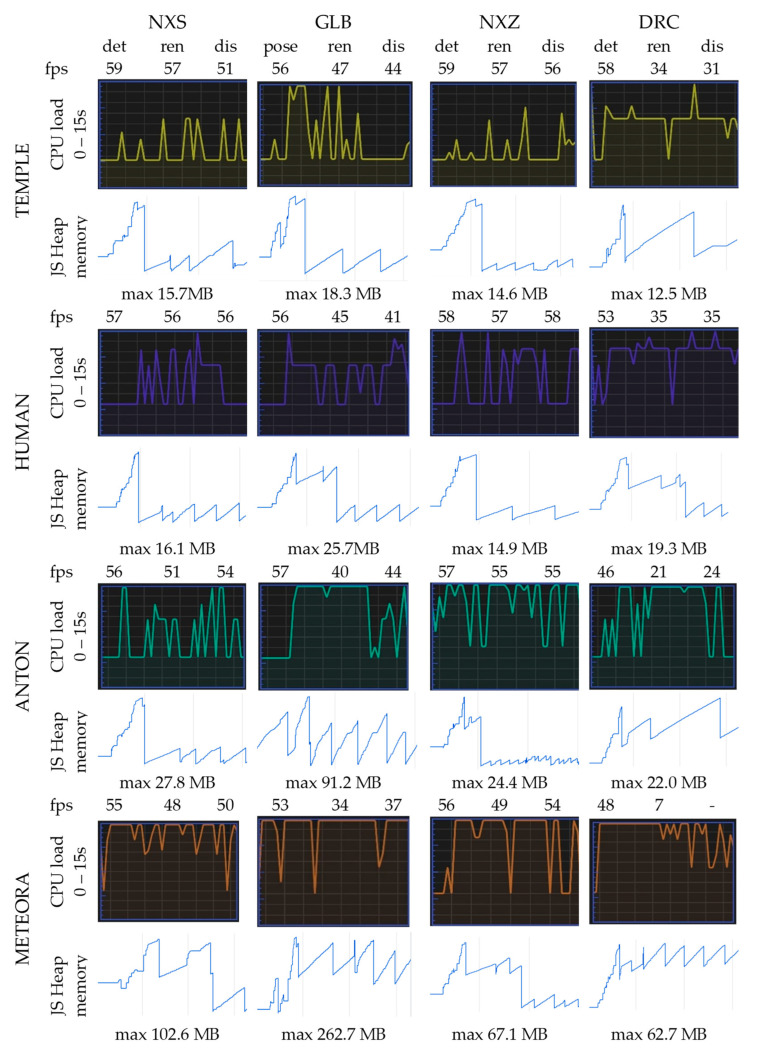
Average values of fps, CPU usage and JS memory heap on UI thread at marker detection (det), rendering (ren) and final display (dis) for NFT-based AR.

The computing and rendering performance, including frame rates, CPU usage and JS memory distribution, are measured, along with the underlying functions, objects and events that are initialized, triggered or run during critical phases. The AR activity is traced for a time period of 15 s for each rendering technique and for each model under 25 mbps/s access. Figure 11 presents the average values on the UI thread at marker detection (det), rendering (ren) and final display (dis). A seamless AR experience requires a stable frame rate of 40–60 fps, which equals a 16.6 msec duration of capturing, processing, rendering and visualizing. Thus, a fixed frame rate of 60 fps is set. If the rendering time exceeds this limit, a frame drop occurs.

The Nexus.js scheme manages to deliver an average stable number of 55 fps. This indicates that it effectively manages the streaming of consecutive data patches from loading to draw time without causing frame drops. It also significantly reduces the computational burden on the CPU. The overall CPU utilization during the camera thread activity is approximately 21% less than glb (in the case of NXS) and 46% less than Draco (in the case of NXZ). The advantage of multi-resolution formats lies in their ability to prioritize and load only the necessary data chunks based on the viewer’s perspective or the current level of detail required. This approach optimizes the rendering process by dynamically loading and rendering only the relevant parts of the model, which can reduce the memory footprint on the JS heap. The increased workload of Draco decompression leads to high overhead, causing latency issues, even in case of the color per vertex Temple model. Moreover, it degrades the user experience with stuttering, a low resolution in textures and a simplified geometry. During the loading of the compressed mesh, re-quantization of the vertex data is performed to match the desired precision. This re-quantization process may introduce quantization errors that affect the visual fidelity of the mesh. The prototype’s tracking accuracy seems to be also affected, leading to errors in object placement and recognition on pose estimation and rendering updates. Based on the inspection of the graphics rendering thread, it can be stated that the maximization of the compression ratio is applied at the expense of disturbing the vertex and index order. This means that the compressed mesh of Meteora may not be as efficient to render on the GPU compared to the original uncompressed mesh. However, in comparison with glb, it is more efficient during the detection and matching of key points and suitable for the AR processing pipeline.

The JS heap represents the portion of memory where JavaScript objects, variables and data structures related to the AR session, including computing and graphics processing, are stored. It becomes large or fragmented in the case of glb models and especially Anton and Meteora, causing performance issues such as frame rate drops and visual degradation. While the Anton and Meteora models are visualized with high fidelity, they appear jumpy and out of sync with the real-world environment. The root cause may be the increased memory allocation and deallocation times as the entire model is typically loaded at once. This means that all the geometry, textures and other associated data are loaded into the memory before the rendering process begins, and it results in a slower startup time compared to multi-resolution formats.

#### 5.3.4. GPU Load

**Figure 12:**
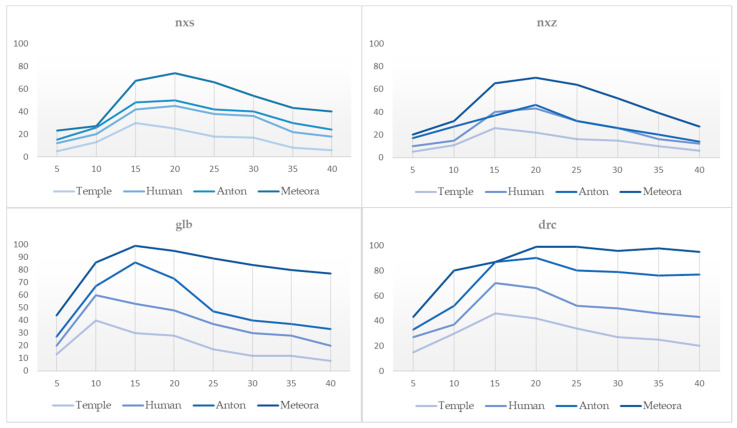
GPU load during NFT AR tracking for each 3D model captured for 40 s.

To further investigate potential bottlenecks that cause low frame rates during the loading of models, the frame rendering timeline is examined for NFT mode. Figure 12 illustrates the GPU load on the device in real time per model. The GPU load percentage remains relatively stable, with minor fluctuations throughout the time intervals for the Temple and Human models across the different methods. In the case of the Meteora model, the maximum rendering queue utilization does not exceed 74%. The high GPU usage is normal in proportion to the recorded CPU load and fps. Moreover, the GPU memory types are efficiently transferred, such as the texture coordinates for the models of Human and Meteora and other relevant bindings.

View-dependent rendering minimizes overdraw by reducing the number of unnecessary pixel calculations during rasterization, the relative wasted computations and memory garbage. Moreover, it optimizes fragment processing by avoiding redundant calculations. By re-ordering the final triangles based on the current view direction, the renderer minimizes the number of fragments that need to be processed, thereby reducing the overall workload on the GPU. As the complexity of rendering effects and the level of detail in the AR scene increase, the pixel shader operations become more computationally demanding. In comparison with DRC, NXZ uses less than 44% of the GPU resources for the Meteora model and 42% for the Anton model. By minimizing overdraw and avoiding redundant fragment processing, view-dependent rendering helps to reduce the workload on the pixel shaders over time, resulting in reduced GPU utilization and improved overall performance (faster rendering times and improved frame rates). The NXS technique tends to utilize fewer GPU resources compared to common glb rendering and their difference ranges from 3.875% to 34.375% across the various models. The GPU load percentage increases significantly over time during Draco-based loading, suggesting a higher load on the GPU as time progresses. This high GPU utilization indicates that the prototype is pushing the limits of the device’s hardware and, as expected, the GPU runs out of resources (OOM error) shortly after 15 s of operation. It must be noted that the effective organization and ordering of the index data, performed during the glb models’ conversion, reduces redundant calculations on the GPU during the vertex processing stage. Thus, despite the size and complexity of the triangles of the Meteora model, the GPU load remains below 95%. This optimization is particularly important for complex and detailed models, where irregularities can potentially place pressure on the GPU.

## 6. Conclusions

The rendering tasks in web AR face an inefficient runtime environment due to the size and complexity of 3D models in conjunction with the limited computational capabilities of mobile devices. The presented work demonstrates the potential of the NXS and NXZ multi-resolution structures of the Nexus.js library in the context of web AR for 3D overlays of varying size, complexity and sources. A prototype that supports two types of AR, NFT-based and location-based, is developed with the open-source libraries of AR.js and Three.js. The user interface of the prototype enables switching between AR sessions and real-time adjustments of the position, rotation, scale, FOV and camera distance for AR overlays. The developed extension of AR.js emphasizes the cluster-based view-dependent visualization during AR session and geometric instancing. These methods are integrated into a Three.js wrapper for runtime rendering on a set of four meshes that vary in complexity, size and vertex/index data. The performance evaluation assesses their efficiency and compares them with common glTF rendering and Draco compression/decompression techniques. The multi-resolution formats of NXS and NXZ achieve fast startup times and the progressive refinement of models, resulting in a smoother and more immersive user experience. A 3D overlay of 56 K vertices and a size of 53 Mb is instantly visualized, while it takes less than 4 s to display a mesh of 362 Mb with relative positioning, albeit at a low resolution, which is refined progressively. The performance analysis showcases the efficacy of the view-dependent Nexus rendering scheme and its potential to serve as a lightweight AR format for large, irregular and textured 3D data derived from photogrammetric and computer vision procedures. To summarize, the following aspects of the presented work showcase its significance in advancing the field of web AR:

- The introduction of multi-resolution NXS and NXZ formats for the efficient handling of large, irregular and textured 3D data in web AR applications;
- The development of a prototype that seamlessly integrates cluster-based view-dependent rendering using Nexus.js with the AR.js and Three.js libraries and applies instancing for pose estimation parameters;
- The comparison and evaluation of the proposed multi-resolution scheme against traditional glTF rendering and Draco decompression techniques;
- Superior performance demonstrated by faster rendering times, reduced CPU and GPU consumption and excellent AR experiences without compromising visual quality;
- A contribution to the progress of web AR by providing a comprehensive and efficient solution for the rendering of high-resolution 3D overlays, as well as by enhancing the visualization and real-time performance in augmented reality experiences.

The evaluation phase sheds light on potential areas for improvement, such as the examination of the impact of additional parameters such as lighting conditions, camera distortion, occlusion and noise. Further tests should be executed with high scene diversity, other combinations of image matching algorithms and pattern objects and for both indoor and outdoor scenarios. In the case of location-based AR, drift and IMU tracking errors accumulated over time in relation to translation, rotation and distance from the camera could be taken into account. The current prototype can be optimized with GPU-based parallelism for acceleration during feature detection and pose estimation, as well as cloud-based rendering. Further work involves the integration and evaluation over a general set of mesh streaming techniques and other 3D structures, such as parallel batched–dynamic data structures.

## Figures and Tables

**Table 1:** Format, size and geometric attributes of the 3D models used as AR overlays, before and after their conversion into multi-resolution structures.

	Original Data					NXS	NXZ
	Format	Size	Faces	Vertices	Color	Size	
Temple	ply	13 MB	574,400	287,257	per vertex	19 MB	2.8 MB
Human	obj	65 MB	999,970	506,024	UV texure	43.5 MB	9.8 MB
Anton	ply	304 MB	12,647,722	63,99,543	per vertex	434 MB	60 MB
Meteora	ply	962 MB	47,913,292	24,201,575	UV texture	1.6 GB	241 MB

**Table 2:** NXS conversion options with their values for each mesh.

	NXS Convertion Options				
	Node Faces	Decimation	Scaling	Adaptive	Color
Temple	20,500	quadric	0.5	0.333	per vertex
Human	70,000	quadric	0.5	0.333	original textures
Anton	100,000	quadric	0.5	0.333	per vertex
Meteora	250,000	quadric	0.5	0.233	original textures

**Table 3:** NXZ compression options with their values for each mesh.

	NXZ Compression Options		
	Vertex Quantization	Quantization Factor	Texture Compression
Temple	8	0.2	-
Human	8	0.4	0.80
Anton	5	0.1	-
Meteora	5	0.1	0.80

**Table 4:** DRACO compression options, encoding time and final size for each mesh.

	DRACO Compresion Options			Results	
	Compression Ratio	Quantization	Texture Precision	Encoding Time	Size
Temple	0	9	-	958 ms	790 KB
Human	4	9	20	454 ms	540 KB
Anton	4	9	-	22,109 ms	13.2 MB
Meteora	8	12	30	96,178 ms	63.8 MB

**Table 5:** glb vertex quantization options, simplification ratio and final size for each mesh.

	Vertex Quantization Bits			Simplification Ratio	Results
	Positions	Normals	Colors		Size
Temple	14	6	14	1	14.3 MB
Human	14	6	-	1	28.8 MB
Anton	16	6	14	1	321.7 MB
Meteora	16	6	-	0.7	1.2 GB

**Table 6:** Mean (ave) values in seconds and standard deviation (%) of time profiling for multi-resolution and common glTF-based rendering performed for each model.

NFT	Temple				Human				Anton				Meteora			
	nxs		glb		nxs		glb		nxs		glb		nxs		glb	
	*ave*	*dev*	*ave*	*dev*	*ave*	*dev*	*ave*	*dev*	*ave*	*dev*	*ave*	*dev*	*ave*	*dev*	*ave*	*dev*
Rendering	0.91	0.85	1.66	0.81	1.03	0.92	2.03	0.77	2.56	0.92	4.36	0.72	3.68	0.85	7.63	0.68
Display	1.53	0.87	2.74	0.79	1.72	0.78	3.97	0.80	3.89	0.83	6.98	0.82	**4.80**	0.76	10.52	0.76
Location-Based	Temple				Human				Anton				Meteora			
	nxs		glb		nxs		glb		nxs		glb		nxs		glb	
Rendering	0.89	0.95	1.38	0.78	0.98	0.83	1.94	0.77	2.30	0.88	4.01	0.74	3.72	0.85	7.60	0.68
Display	1.42	0.89	2.30	0.87	1.57	0.93	3.95	0.89	2.91	0.92	6.35	0.89	**4.51**	0.95	10.24	0.75

**Table 7:** Mean (ave) values in seconds and standard deviation (%) of time profiling for compressed multi-resolution NXZ and DRC rendering for each model.

NFT	Temple				Human				Anton				Meteora		
	nxz		drc		nxz		drc		nxz		drc		nxz		drc
	*ave*	*dev*	*ave*	*dev*	*ave*	*dev*	*ave*	*dev*	*ave*	*dev*	*ave*	*dev*	*ave*	*dev*	*-*
Decoding	0.46	0.94	2.54	0.87	0.58	0.91	3.45	0.78	1.33	0.80	16.28	0.70	1.99	0.88	-
Rendering	0.59	0.90	4.01	0.70	1.06	0.88	5.98	0.79	2.04	0.79	20.06	0.61	3.57	0.77	-
Display	1.16	0.82	4.68	0.73	1.85	0.84	6.55	0.76	3.21	0.83	22.30	0.56	**4.23**	0.85	-
Location-Based	Temple				Human				Anton				Meteora		
	nxz		drc		nxz		drc		nxz		drc		nxz		drc
Decoding	0.47	0.92	2.52	0.90	0.58	0.89	3.46	0.74	1.32	0.82	15.99	0.66	1.99	0.76	-
Rendering	0.49	0.89	3.77	0.73	1.00	0.88	5.25	0.84	1.86	0.89	19.13	0.69	3.18	0.73	-
Display	1.22	0.93	4.10	0.91	1.39	0.90	5.93	0.86	2.45	0.93	21.54	0.82	**3.95**	0.89	-

## Data Availability

The data presented in this study are openly available in Zenodo at 10.5281/zenodo.8071718.
